# A Critical Perspective on *Helicobacter pylori* Treatment Strategies: Are Levofloxacin-Based Regimens Truly Suitable as First-Line Therapy?

**DOI:** 10.5152/tjg.2026.25791

**Published:** 2026-03-23

**Authors:** Ramazan Dertli, Mehmet Asil

**Affiliations:** Division of Gastroenterology, Department of Internal Medicine, Necmettin Erbakan University Meram School of Medicine, Konya, Türkiye

Dear Editor,

I read with great interest the article by Kanat Unler et al, entitled “Best Treatment Options for Severe *Helicobacter pylori* Infections.”^1^ *Helicobacter pylori* infects more than half of the global population and invariably causes chronic gastritis, which may lead to serious complications such as peptic ulcer disease, gastric adenocarcinoma, and gastric Mucosa-associated lymphoid tissue (MALT) lymphoma.^2^^,^^3^ The Maastricht VI/Florence Consensus Report has emphasized the critical role of *H. pylori* eradication in achieving the goal of reducing or eliminating gastric cancer–related mortality.^2^

In their study, Kanat Unler et al reported *H. pylori* eradication rates of 74.8%, 85.3%, and 74.8% with bismuth-containing sequential clarithromycin therapy, bismuth-containing sequential levofloxacin therapy, and bismuth quadruple therapy, respectively. In a recently conducted study from our country,^4^ RMAB (rabeprazole, metronidazole, amoxicillin, and bismuth) and EMTB (esomeprazole, metronidazole, tetracycline, and bismuth) regimens were used as first-line therapy, achieving per-protocol eradication rates of 66.9% and 68.5%, respectively. In the same study, rabeprazole, tetracycline, metronidazole, amoxicillin, and bismuth (RTAMB) and esomeprazole, tetracycline, amoxicillin, metronidazole, and bismuth (ETAMB) quintuple regimens were applied as second-line therapy, yielding per-protocol eradication rates of 65.6% and 67.6%, respectively. Increasing resistance rates to key antibiotics used in *H. pylori* treatment, such as clarithromycin and levofloxacin, have been reported both globally and in Türkiye.^5^^,^^6^ In Türkiye, clarithromycin resistance has been reported at 26.7% and levofloxacin resistance at 19.6%.^6^ While antimicrobial resistance continues to represent a major global health challenge, recommending levofloxacin-based regimens as first-line therapy based on the results of the study should be interpreted with caution in regions with high resistance rates. Although the reported eradication rates appear high, increasing levofloxacin resistance may lead to unfavorable and potentially irreversible consequences in the long term.

International guidelines primarily recommend bismuth-based quadruple therapies as first-line treatment.^2^^,^^5^ According to the Maastricht VI/Florence Consensus Report, following the failure of Proton pump inhibitors (PPI)–clarithromycin–amoxicillin triple therapy, second-line options include bismuth-containing quadruple therapy, fluoroquinolone-containing quadruple (or triple) therapy, or high-dose PPI–amoxicillin dual therapy.^2^ On the other hand, the American College of Gastroenterology Clinical Guideline recommends levofloxacin-based triple therapy only in previously treated patients with persistent *H. pylori* infection and known levofloxacin-susceptible strains and in cases where optimized bismuth quadruple or rifabutin-based triple therapies have already been used or are unavailable.^5^

Taken together, these data suggest that, particularly in regions with high levofloxacin resistance, levofloxacin-based regimens may be more appropriately considered as second-line or rescue therapy options.

## Data Availability

The data that support the findings of this study are available on request from the corresponding author.
